# Genomic analysis of bacteria in the Acute Oak Decline pathobiome

**DOI:** 10.1099/mgen.0.000240

**Published:** 2019-01-08

**Authors:** James Doonan, Sandra Denman, Justin A. Pachebat, James E. McDonald

**Affiliations:** ^1^​ School of Biological Sciences, Bangor University, Bangor, UK; ^2^​ Forest Research, Centre for Forestry and Climate Change, Farnham, UK; ^3^​ Institute of Biological, Environmental and Rural Sciences, Aberystwyth University, Aberystwyth, UK

**Keywords:** Acute Oak Decline (AOD), *Brenneria goodwinii*, necrosis, pathobiome, phytopathogens

## Abstract

The UK’s native oak is under serious threat from Acute Oak Decline (AOD). Stem tissue necrosis is a primary symptom of AOD and several bacteria are associated with necrotic lesions. Two members of the lesion pathobiome, *Brenneria goodwinii* and *Gibbsiella quercinecans*, have been identified as causative agents of tissue necrosis. However, additional bacteria including *Lonsdalea britannica* and *Rahnella* species have been detected in the lesion microbiome, but their role in tissue degradation is unclear. Consequently, information on potential genome-encoded mechanisms for tissue necrosis is critical to understand the role and mechanisms used by bacterial members of the lesion pathobiome in the aetiology of AOD. Here, the whole genomes of bacteria isolated from AOD-affected trees were sequenced, annotated and compared against canonical bacterial phytopathogens and non-pathogenic symbionts. Using orthologous gene inference methods, shared virulence genes that retain the same function were identified. Furthermore, functional annotation of phytopathogenic virulence genes demonstrated that all studied members of the AOD lesion microbiota possessed genes associated with phytopathogens. However, the genome of *B. goodwinii* was the most characteristic of a necrogenic phytopathogen, corroborating previous pathological and metatranscriptomic studies that implicate it as the key causal agent of AOD lesions. Furthermore, we investigated the genome sequences of other AOD lesion microbiota to understand the potential ability of microbes to cause disease or contribute to pathogenic potential of organisms isolated from this complex pathobiome. The role of these members remains uncertain but some such as *G. quercinecans* may contribute to tissue necrosis through the release of necrotizing enzymes and may help more dangerous pathogens activate and realize their pathogenic potential or they may contribute as secondary/opportunistic pathogens with the potential to act as accessory species for *B. goodwinii*. We demonstrate that in combination with ecological data, whole genome sequencing provides key insights into the pathogenic potential of bacterial species whether they be phytopathogens, part-contributors or stimulators of the pathobiome.

## Data Summary

1. *Gibbsiella quercinecans* FRB124, BioSample SAMN05732392. Genome assembly deposited in GenBank; accession number MJLV 00000000. Illumnia MiSeq data have been deposited in the Sequence Read Archive; experiment SRX2141032.

2. *Gibbsiella quercinecans* FRB97, BioSample SAMN05732390. Genome assembly deposited in GenBank; accession number MJLU00000000. Illumnia MiSeq data have been deposited in the Sequence Read Archive; experiment SRX2141032.

3. *Brenneria alni* NCPPB 3934. BioSample SAMN05733147. Genome assembly deposited in GenBank; accession number MJLZ00000000. Illumnia MiSeq data have been deposited in the Sequence Read Archive; experiment SRX2141032.

4. *Brenneria goodwinii* FRB171, BioSample SAMN05732394. Genome assembly deposited in GenBank; accession number MJLY00000000. Illumnia MiSeq data have been deposited in the Sequence Read Archive; experiment SRX2141032.

5. *Gibbsiella quercinecans* N78, BioSample SAMN05732390. Genome assembly deposited in GenBank; accession MJLW00000000. Illumnia MiSeq data have been deposited in the Sequence Read Archive; experiment SRX2141032.

6. *Brenneria goodwinii* FRB141, BioSample SAMN05732419. Genome assembly deposited in GenBank; accession MJLX00000000. Illumnia MiSeq data have been deposited in the Sequence Read Archive; experiment SRX2141032.

7. *Brenneria salicis* DSM30166, BioSample SAMN04999998. Genome assembly deposited in GenBank; accession MJMA00000000. Illumnia MiSeq data have been deposited in the Sequence Read Archive; experiment SRX2141032.

8. *Rahnella variigena* CIP105588T, BioSample SAMN07554573. PacBio RS II run has been deposited in Sequence Read Archive: SRX3145876. Genome assembly and motif summary files have been deposited in GenBank; accession NSDJ00000000.

9. *Lonsdalea britannica* 477, BioSample SAMN07554530. PacBio RS II run has been deposited in Sequence Read Archive: SRX3131452. Genome assembly and motif summary files have been deposited in GenBank; accession CP023009.

10. All data are held under NCBI BioProject PRJNA342025.

**Impact Statement**

Acute Oak Decline (AOD) is a complex decline disease and a serious threat to native oak in the UK. Recently, a bacterial pathobiome (a microbiome associated with disease) has been shown to cause tissue necrosis on oak logs, and two bacterial species, *Brenneria goodwinii* and *Gibbsiella quercinecans*, are key necrotic agents. Transcriptome analysis of field material suggests that *B. goodwinii* is the key necrotizing phytopathogen within AOD, whilst *G. quercinecans* and other pathobiome members have an as yet unspecified role in the disease. Comparative genomic analysis of AOD pathobiome members enables assessment of host–microbe and microbe–microbe interactions in AOD. Here we compare the whole genome sequences of *G. quercinecans*, *B. goodwinii* and other members of the AOD lesion pathobiome against known phytopathogens and non-pathogens. Genome analysis suggests that *B. goodwinii* and *Lonsdalea britannica* (the latter a species occasionally isolated from AOD lesions) are potential primary pathogens within a predisposed tree, whereas other pathobiome members including *G. quercinecans* may only contribute to tissue necrosis through the release of necrotizing enzymes leading to the proposal that they are generalists that may help more dangerous pathogens activate and realize their pathogenic potential or that they may contribute as secondary/opportunistic pathogens with the potential to act as accessory species for *B. goodwinii*. Therefore, we provide supportive evidence that AOD is caused by an interactive bacterial pathobiome, and thus add to the expanded concept of tree diseases caused by polymicrobial complexes. This work provides important insights into the functional and ecological roles of several key members of the complex AOD pathobiome, associated with tissue necrosis, and highlights the importance of characterizing host–microbe and microbe–microbe interactions in a complex pathosystem.

## Introduction

Acute Oak Decline (AOD) is a recently described decline-disease in Britain affecting both native species of oak, *Quercus robur* L. (pedunculate oak) and *Quercus petraea* (Matt.) Liebl. (sessile oak) [1]. AOD is increasing in incidence and distribution, occurring predominantly in the south-east and Midlands of England, spreading from east to west, and has now been recorded in south Wales [2, 3]. Consistent with other complex declines, the causative agents of AOD are biotic and abiotic, although no complete definition of the pathogenic potential of the biotic agents involved in the weeping stem and inner-bark disease symptoms, and their function is yet available. This is in part due to the complexity of the decline syndrome [1, 4]. Two recently described bacteria, *Gibbsiella quercinecans* [5] and *Brenneria goodwinii* [6], have been identified as causal agents of necrotic lesions on AOD-affected trees [3]. *B. goodwinii* is highly abundant and dominated the AOD lesion microbiome in previous studies; *G. quercinecans* was consistently present in the AOD lesion microbiome [3, 7]. Furthermore, the ability of both species to cause tissue necrosis after direct inoculation onto non-symptomatic oak logs and trees has been documented [3]. A third bacterial species, *Rahnella victoriana*, was also abundant in the lesion microbiome but has, as yet, an undefined role [3]. Currently, the specific mechanisms through which necrotic lesions occur, and the role of individual lesion microbiota as components of a complex pathobiome, is unknown.

A key challenge in analysing and assigning functional roles in complex pathobiomes is separating pathogens from symbionts. Virulence mechanisms used by bacterial phytopathogens to target plants can be uncovered using whole genome sequencing (WGS) [8, 9]. Increasingly, WGS is the first step in the process of seeking evidence for pathogenic potential of putative bacterial pathogens of undiagnosed disease [10, 11]. New virulent strains of characterized pathogens (i.e. organisms with well-defined virulence mechanisms such as *Pseudomonas syringae* pv. *syringae*) can be rapidly identified by detecting unaligned stretches of DNA suggesting gene acquisition, loss or duplication [12, 13]. For novel bacterial putative pathogens which lack a characterized strain, the situation is more complex. In this scenario, bacteria are isolated and sequenced, and evolutionary conserved virulence homologues are identified through sequence similarity searches, which reveal putative pathogenicity mechanisms and gene targets for further testing [14]. Comparative genomics therefore allows an analysis of idiosyncratic pathogen biology, providing an evidence-based approach to identify the mechanisms of pathogenicity in individual species and ultimately prescribes some of the tools to control disease [15].

Despite advances in genomics, the goal of certifying pathogenicity from genomics alone has not been realized, and cannot yet replace empirical functional evidence [8, 14, 16, 17]. Linking the presence of functional virulence genes with observed pathogenic activity is therefore a crucial step in proof of pathogenicity [3] and the subsequent identification and characterization of specific virulence mechanisms associated with disease. Within bacterial phytopathogens, it is accepted that direct virulence factors include plant cell wall degrading enzymes (PCWDEs) (particularly pectinases, but also cellulases, hemicellulases, tannases), a functional type III secretion system (T3SS) and associated effectors [18–21]. Apparent anomalies, such as the presence of virulence factor homologues in non-pathogenic symbionts, are a barrier to effective delineation of functional roles in pathobiomes [14]. Here, pathogens are symbionts *sensu stricto*, as they live in close proximity to the host and have biological interactions [22]; symbionts are classified as biological organisms that are not known to cause disease, i.e. mutualists, commensals and parasites, but not pathogens. Symbionts encoding virulence genes appear incongruous, but these genes are often remnants from evolutionary history that are being purged from the genome as they are no longer required [23, 24], or are utilized by micro-organisms for symbiotic interactions with the host. Examples of virulence genes most commonly found in pathogens but also found in symbionts include the T3SS [25, 26], toxins [27] and invasion genes [28]. Similarly often overlooked is the necessity for symbionts to use genes associated with virulence such as pili or flagella, simply to colonize the host, where they assist non-pathogenic occupation of the symbionts favoured niche [26]. Thus, clearly some symbiotic bacteria have pathogenicity genes that can be dormant or superfluous, or used in a benign way to enable colonization of a host or substrate.

A second challenge for the clear demarcation of a pathogen within functional and genomic analyses relates to hemibiotrophic pathogens, which can exist as lifelong asymptomatic biotrophs and have a mixed genomic repertoire enabling them to exist as biotrophs and latent pathogens as conditions dictate [26]. A third challenge is accounting for saprophytes that usually feed on decaying matter, but with high inoculum can cause disease in a healthy host, or indeed for those bacteria that can switch between saprophytic and pathogenic roles [29]. These natural variations make rigorous classification of microbial ecofunctionality of little value and restrict the ability of bioinformatic approaches to separate symbionts from pathogens [14]. A more rigorous approach is to measure the pathogenic potential of a bacterium without pre-supposing a single outcome, but rather basing ecofunctional classification on an interactive outcome which depends on inherent genetic potential as well as interactions at the host–bacteria interface [30].

Koch’s postulates are the central dogma of disease aetiology for novel pathogens and continue to be a diagnostic requirement [31]. Previously, a contemporary adaptation of Koch’s postulates that combined modern molecular technologies with traditional microbial pathology experiments provided evidence that AOD symptoms were caused by a complex pathobiome of multi-organism disease-causing agents, with *G. quercinecans* and *B. goodwinii* as two causative agents of tissue necrosis within AOD [3]. Furthermore, a multi-omic study revealed the metagenomic enrichment and metatranscriptomic upregulation of virulence genes aligned against *G. quercinecans* but particularly *B. goodwinii*, and proteome data revealed upregulated phytopathogenic proteins in AOD field lesions [7]. Pathobiome-mediated disease is becoming increasingly accepted within clinical and phytological research as a biological reality of disease causation [32, 33]. Here, we investigate genomic signatures of pathogenicity within key members of the polymicrobial consortia isolated from necrotic lesions of trees affected with AOD, and investigate the role of those that lack clear pathogenic signatures and have an unknown role within the lesion pathobiome [17, 24, 26]. Furthermore, we describe genome-encoded virulence factors that may contribute to tissue necrosis within AOD, providing key linkages to previous meta-transcriptomic work [3, 7].

## Methods

### Maintenance of bacterial strains

*G. quercinecans* strains FRB97 and FRB124, *B. goodwinii* strains FRB141 and FRB171, *L. britannica* strain 477 and *R. victoriana* strain BRK18a were isolated by Forest Research (Alice Holt Lodge, Surrey, UK) from oak trees affected with AOD (Tables 1 and 2). *Rahnella variigena* strain CIP105588T was obtained from a culture collection and represented *R. variigena* strains previously isolated from necrotic lesions on AOD-affected trees. *Brenneria alni* NCPPB3934 and *Brenneria salicis* DSM 30166 were also obtained from culture collections. Bacterial strains were previously identified to species level through multi-locus sequence analysis and DNA–DNA hybridization [5, 6, 34, 35]. Isolates were stored in 40 % glycerol stocks at −80 °C and maintained on nutrient agar (Oxoid) at 20 °C.

**Table 1.** Genome metrics of bacterial isolates identified from necrotic lesions of AOD-affected trees

Organism (accession)	Family	Origin	No. of contigs	No. of genes (gene density %)	Degree of orthology (degree of virulence orthology)	Chromosome size (bp)/GC content (mol%)
*Gibbsiella quercinecans* FRB97 (CP014136)	*Enterobacteriacae*	Hoddesdon Park, UK [5]	1	5125 (86.9)	21 (16)	5 548 506 (56)
*Gibbsiella quercinecans* FRB124 (MJLV00000000)	*Enterobacteriacae*	Outwood, UK [5]	90	4852 (86.6)	23 (16)	5 469 793 (56)
*Gibbsiella quercinecans* N78 (MJLW00000000)	*Enterobacteriacae*	Burgos, Spain [5]	129	5202 (86.4)	2 (0)	5 693 731 (56)
*Brenneria goodwinii* FRB141 (CP014137)	*Pectobacteriacae*	Outwood, UK [6]	1	4625 (85.8)	20 (20)	5 281 917 (51)
*Brenneria goodwinii* FRB171 (MJLY00000000)	*Pectobacteriacae*	Gorse Covert, UK [6]	128	4881 (86.1)	18 (20)	5 377 922 (53)
*Lonsdalea britannica* 477 (CP023009)	*Pectobacteriacae*	Surrey, UK [34]	1	3801 (87.2)	15 (20)	4 015 589 (55)
*Rahnella variigena* CIP105588T (NSDJ00000000)	*Yersiniaceae*	Culture collection (representative strain) [35]	2	5187 (89.7)	21 (20)	5 499 108 (52)
*Rahnella victoriana* BRK18a (MAEN01000001)	*Yersiniaceae*	Brock Hampton, UK [35]	2	5230 (90.2)	23 (20)	5 563 295 (53)

**Table 2.** Genome metrics of bacterial contigs/plasmids (replicons) downloaded from NCBI and two phytopathogenic *Brenneria* species sequenced in this study

Organism (accession)	Family	Origin (information presented where available)	Contigs/plasmids	No. of chromosomal genes (gene density %)	Degree of orthology (degree of virulence orthology)	Chromosome size (bp) (G+C content, mol%)	Reference
*Agrobacterium tumefaciens* Ach5 (CP011246)	*Rhizobiaceae*	Yarrow (*Achillea ptarmica*), Contra Costa County, CA, USA	4/2	2795 (circular) (90.3) 1915 (linear) (91.8)	16 (13)	2 833 887 (58.8) (circular) 2 095 752 (58.6) (linear)	[75]
*Azospirillum brasilense* Sp7 (CP012914)	*Rhodospirillaceae*	*Digitaria eriantha*, Brazil	6/5	2833 (89.4)	20 (2)	3 005 726 (68.2)	–
*Bacillus licheniformis* ATCC 14580 (NC_006270)	*Bacillaceae*	Culture collection	1	4479 (90.3)	0 (0)	4 222 597 (46.2)	[76]
*Brenneria alni* NCPPB 3934 (MJLZ00000000)	*Pectobacteriacae*	Italian alder (*Alnus cordata*), Italy. Causative agent of bark canker [36]	132/-	4013 (86.9)	16 (20)	4 127 267 (52.4)	This study
*Brenneria goodwinii* OBR1 (CGIG00000000)	*Pectobacteriacae*	–	1	4835 (88.3)	20 (20)	5 350 059 (53.1)	–
*Brenneria salicis* DSM30166 (MJMA00000000)	*Pectobacteriacae*	Willow (*Salix alba* var. *caerulea*), UK. Causative agent of watermark disease [37]	106/-	3781 (86.4)	16 (20)	3 929 937 (52.1)	This study
*Dickeya dadantii* 3937 (NC_014500)	*Pectobacteriacae*	–	1	4513 (87.8)	19 (21)	4 922 802 (56.3)	[77]
*Erwinia amylovora* CFBP1430 (NC_013961)	*Erwiniaceae*	European isolate	2/1	3566 (87.6)	15 (19)	3 805 573 (53.6)	[78]
*Erwinia billingiae* Eb661 (NC_014306)	*Erwiniaceae*	–	3/2	4784 (90.1)	22 (19)	5 100 167 (55.2)	[79]
*Gluconacetobacter diazotrophicus* PA1 5 (NC_011365)	*Acetobacteraceae*	Culture collection	2/1	3666 (91.2)	21 (16)	3 887 492 (66.4)	[80]
*Herbaspirillum seropedicae* Z67 (CP011930)	*Oxalobacteraceae*	Maize/sorghum/rice, Rio de Janeiro, Brazil	1	4850 (90)	23 (16)	5 509 723 (63.4)	–
*Methylobacterium mesophilicum* SR1.6/6 (ANPA01000003)	*Methylobacteriaceae*	*Citrus sinensis,* Brazil	29/-	6050 (86.4)	7 (0)	6 214 449 (69.5)	[81]
*Pectobacterium carotovorum* subsp. *carotovorum* PC1 (NC_012917)	*Pectobacteriacae*	–	1	4461 (89.3)	20 (20)	4 862 913 (51.9)	–
*Pseudomonas syringae* pv. *syringae* B728a (NC_007005)	*Pseudomonadaceae*	Snap bean (*Phaseolus vulgaris*), Wisconsin, USA	1	5356 (90.1)	25 (23)	6 093 698 (59.2)	[82]
*Ralstonia solanacearum* GM1000 (NC_003295)	*Burkholderiaceae*	Tomato	2/1	3525 (89.9)	3 (1)	3 716 413 (67)	[83]
*Rhizobium leguminosarum* bv. *trifolii* WSM1689 (CP007045)	*Rhizobiaceae*	*Trifolium uniflorum*, Naxos, Greece	6/5	4770 (88.6)	7 (2)	4 854 518 (61.1)	[84]
*Rhizobium leguminosarum* bv. *viciae* 3841 (NC_008380)	*Rhizobiaceae*	Plant habitat	7/6	4937 (85)	11 (2)	5 057 142 (61.1)	[85]
*Xanthomonas axonopodis* Xac29-1 (NC_020800)	*Xanthomonadaceae*	–	4/3	4513 (87.6)	15 (16)	5 153 455 (64.8)	–
*Xanthomonas campestris* ICMP 21080 (CP012145)	*Xanthomonadaceae*	Cabbage, Southbridge, New Zealand	1	4333 (87.4)	15 (16)	4 911 121 (65.3)	[86]
*Xanthomonas oryzae* pv. *oryzae* MAFF 311018 (NC_007705)	*Xanthomonadaceae*	–	1	4983 (87.4)	8 (16)	4 940 217 (63.7)	[87]
*Xylella fastidiosa* Hib4 (NZ_CP009885)	*Xanthomonadaceae*	*Hibiscus*, Sao Paulo, Brazil	2/1	2846	0 (4)	2 813 297 (52.7)	–

### Bioinformatic analysis of genome data

Bioinformatic analyses were carried out on SuperComputing Wales, an HPC network, using GNU/Linux Red Hat Enterprise Linux Server release 7.4 (Maipo). A complete list of commands used to perform the below analysis is hosted on GitHub (https://github.com/clydeandforth/MGen.git).

### Genome sequencing using the Illumina MiSeq platform

Two strains of *G. quercinecans*, one strain of *B. goodwinii* and one strain each of *B. alni* (NCPPB 3934) and *B. salicis* (DSM 30166), the latter two both being plant pathogens associated with bleeding stem cankers on alder [36] and willow [37] respectively, were sequenced using the Illumina MiSeq (Tables 1 and 2). A single colony of each strain was selected from cultures streaked on nutrient agar (Oxoid) and inoculated into liquid nutrient broth (Oxoid) and incubated overnight at 28 °C, on a shaking incubator at 100 r.p.m. Total genomic DNA was isolated from the resulting culture using the Genomic II extraction kit (Bioline) following the manufacturer’s protocol. Extracted DNA was quantified using the Qubit fluorometer (Life Technologies). DNA integrity was assessed using 2 % agarose gel electrophoresis. DNA sequencing libraries were prepared using the Illumina Nextera XT DNA protocol (Illumina). Briefly, samples were equalized for an input concentration of 1 ng µl^−1^. DNA was fragmented, tagged (‘tagment’) and appended with adapters using an engineered transposome. The adapters were used as amplification targets for a 20-cycle PCR. During the PCR, target DNA (insert) was amplified, and indexed sequences were added to both ends of the DNA, allowing paired end amplification of the insert. Finally, a further PCR was performed as per the manufacturer’s instructions with the exception that 16 thermal cycles were completed as opposed to 12. Amplicon and insert size were assessed through 2 % agarose gel electrophoresis. Amplified DNA was purified using Agencourt AMPure XP beads (Beckman Coulter) and normalized with library normalization additives. Samples were adjusted to a concentration of 2 nM in 10 mM Tris-HCl and 0.1 % Tween before being heat denatured and added to a single lane of the MiSeq Personal Sequencer (Illumina).

### Post-sequencing quality control

Nextera XT adapter sequences were removed from raw FastQ files containing resultant sequencing reads, using Cutadapt v1.2.1 [38], with the option –O3, which specifies that a minimum of 3 bp must match the adapter sequences before trimming. Sequences were quality trimmed using Sickle v1.2 [39] with a minimum quality score of 20. Reads of fewer than 10 bp were removed.

### Bacterial genome assembly

Bacterial genomic DNA sequences from the Illumina MiSeq were assembled *de novo* using SPAdes v3.0 [40], with k-mer values of 21, 33, 55, 77, 99,121, 143, 165, 187, 209 and 231 for all genomes. *G. quercinecans* FRB124 was assembled into 90 contigs with 92× coverage, *G. quercinecans* N78 assembled into 129 contigs with 75× coverage and *B. goodwinii* FRB171 assembled into 128 contigs with 52× coverage (Table 1).

### Genome sequencing on Pacific Biosciences RSII platform

The whole genomes of *L. britannica* 477 and *R. variigena* CIP105588T were sequenced using the Single Molecule Real-Time (SMRT) technology of the Pacific Biosciences RSII platform (PacBio). A single colony of each isolate was sampled from nutrient agar (Oxoid) and cultured as described above. Total genomic DNA was extracted from an overnight nutrient broth culture using the Gentra Puregene Yeast/Bact. kit (Qiagen) and quantified using a Qubit fluorometer (Life Technologies). DNA integrity was assessed using 1 % agarose gel electrophoresis. DNA libraries were prepared using 20 µg of genomic DNA and sequenced by the Centre for Genomic Research, University of Liverpool, UK, with DNA sheared to approximately 20 kb, and data generated using P6/C4 chemistry and one SMRT cell. Whole genome PacBio assemblies of *G. quercinecans* FRB97, *B. goodwinii* FRB141 and *R. victoriana* BRK18a were generated in a previous study [3].

### Genome assembly of Pacific Biosciences RSII generated data

Our *de novo* genome assembly was performed using the hierarchical genome assembly 3 (HGAP3) workflow [41], incorporating the CELERA assembler. Resultant assemblies produced one contig for *L. britannica* 477 and two contigs for *R. variigena* CIP105588T (Table 1). The *L. britannica* genome had an average coverage of 176×, and the *R. variigena* contigs had average coverages of 173× and 148×. The assemblies were finished using the Quiver consensus polisher, giving mean confidence values (QV) of 48 for all contigs.

### Data availability

All sequence data generated for this study are available under BioProject PRJNA342025.

### Additional whole genome sequence data

Ten bacterial pathogen strains consisting of seven species and three pathovars representing the top ten bacterial plant pathogens [21] and selected symbionts were downloaded from NCBI (Table 2). For comparative purposes the Gram-positive, saprophytic bacterium *Bacillus licheniformis* ATCC14580 was selected as an outgroup in the analysis. These genomes were incorporated into the workflow described below.

### Identification of orthologues

Structural annotations of all genome assemblies for all study organisms (*n*=29) were generated using the Prokka annotation pipeline v1.12 [42]. A shared set of orthologues were identified using OrthoFinder v2.2.7 [43]. Only orthologues shared among two or more genomes were used in subsequent analyses. Orthologues are clustered into groups based on sequence similarity as part of the OrthoFinder workflow, and these clustered groups (which contain multiple orthologues) are called orthogroups. Hereafter, the combined set of clustered orthologues are referred to as orthogroups.

### Virulence gene clustering

Structural gene annotations from Prokka [42] were queried against the Virulence Factor Database (VFDB) [44] accessed on 29 October 2018, using the blastp command within Diamond v0.9.22 [45] with a query cut-off value of 97% and percentage identity greater than or equal to 50. These cut-offs were designed for high sequence identity alignments between bacterial genes and virulence factors. A shared set of virulence orthogroups (*n*=312) among all study organisms (*n*=29) were identified using OrthoFinder v2.2.7 [43]. Only orthogroups shared among two or more genomes were used in subsequent analyses.

### Undirected graph-based visualization of orthogroup networks

**Fig. 1.**
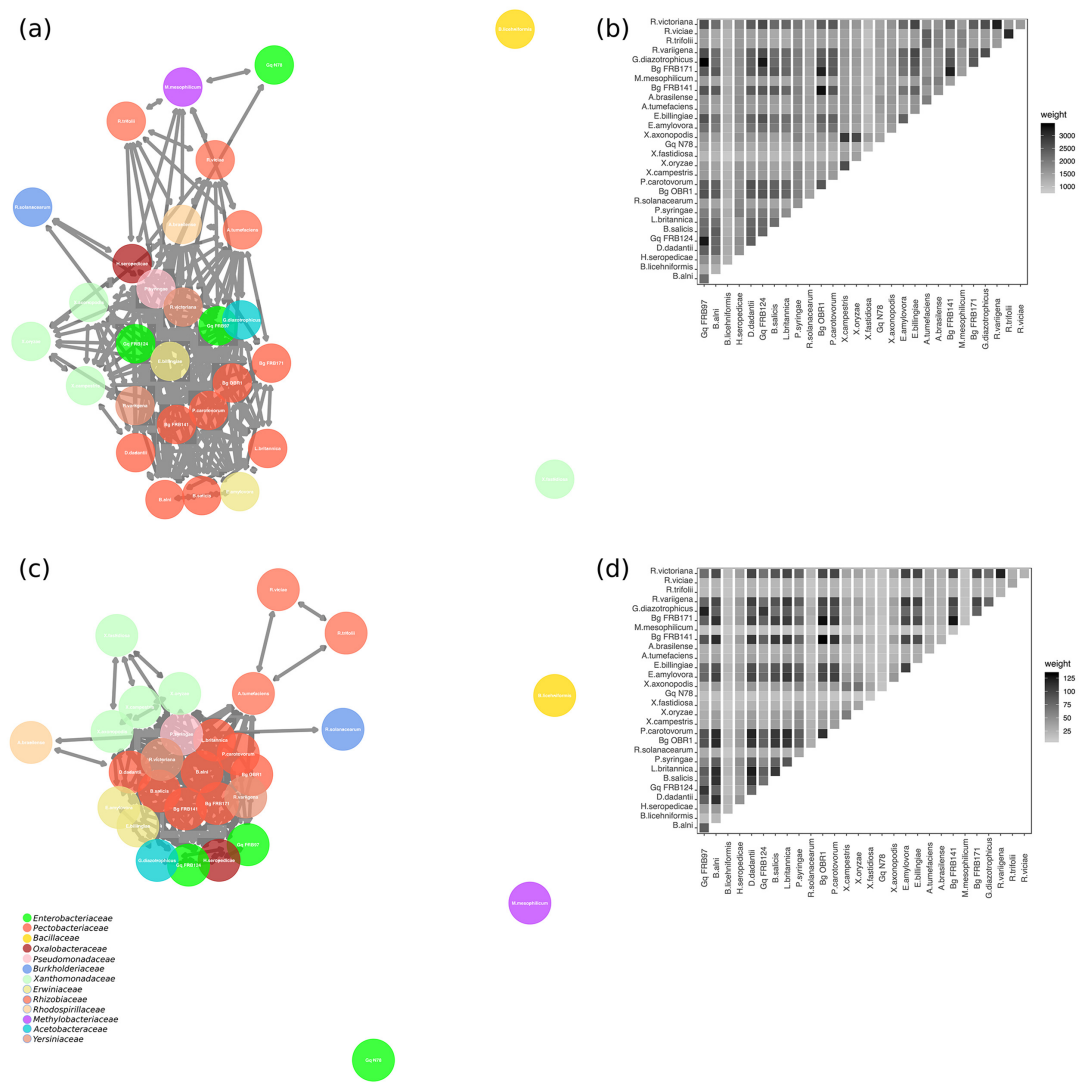
(a) Orthologous clustering network of shared genes amongst study organisms. There were 29 study organisms, 9281 orthogroups and >20 000 orthologues within the orthogroups. Connected nodes show conserved evolution of gene function. Connected edges represent a high number of shared orthologous virulence genes. Nodes are coloured by bacterial family. For full details of all bacteria in this study see Tables 1 and 2. (b) Weighted adjacency matrix of all orthogroups. Pairwise comparisons are shaded in each box. Shading increases are equivalent to increasing edge incidence. (c) Orthologous clustering network of shared virulence genes amongst study organisms. Connected edges represent a high number of shared orthologous virulence genes. Nodes are coloured by bacterial family. For full details of all bacteria in this study see Tables 1 and 2. (d) Weighted adjacency matrix of virulence orthogroups. Pairwise comparisons are shaded in each box. Shading increases are equivalent to increasing edge incidence. Gq=*Gibbsiella* *quercinecans*, Bg=*Brenneria* *goodwinii*.

**Fig. 2.**
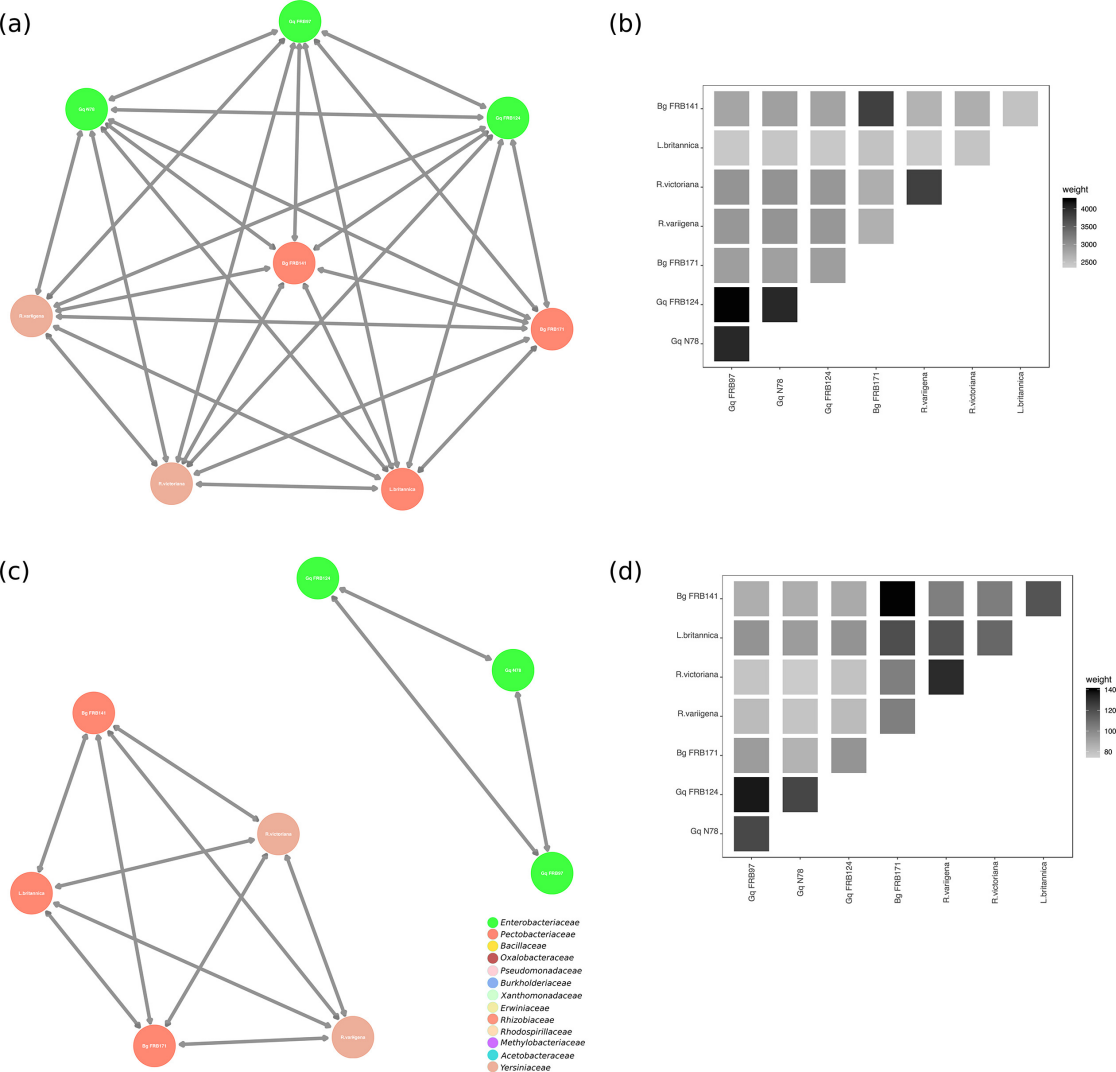
(a) Orthologous clustering network of shared genes amongst organisms isolated from AOD lesions. Connected nodes show conserved evolution of gene function. Connected edges represent a high number of shared orthologous virulence genes. Nodes are coloured by bacterial family. For full details of all bacteria in this study see Tables 1 and 2. (b) Weighted adjacency matrix of orthogroups amongst bacterial isolates from AOD lesions. Pairwise comparisons are shaded in each box. Shading increases are equivalent to increasing edge incidence. (c) Orthologous clustering network of shared virulence genes amongst bacterial isolates from AOD lesions. Connected edges represent a high number of shared orthologous virulence genes. Nodes are coloured by bacterial family. For full details of all bacteria in this study see Tables 1 and 2. (d) Weighted adjacency matrix of orthogroups amongst bacterial isolates from AOD lesions. Pairwise comparisons are shaded in each box. Shading increases are equivalent to increasing edge incidence. Gq=*Gibbsiella* *quercinecans*, Bg=*Brenneria* *goodwinii*.

Undirected graphs (networks) were generated using KinFin v1.0 (Figs 1a, c and 2a, c). Graphs were analysed and visualized using igraph v1.2.2 [46], ggplot2 v3 [47] and GGally v1.4 [47]. The number of orthogroups (*n*=9281) was used to measure nodes (*n*=29 for each genome used in the analysis), degree (i.e. number of incident edges for each node) and the weight of each edge (the sum of the edges). These values provide a measure of shared ancestry among protein coding genes. Edges are visualized as adjoining lines between nodes, with each connecting edge representing one degree. Low weight edges were removed from all graphs (i.e. those edges weighted with a value less than or equal to 1500 in the complete graph, 30 in the virulence orthology graph and 100 in the graphs using AOD isolates only). All graphs were drawn using the Kamada-Kawai Force directed algorithm, with theoretical distance between nodes related to the geometric (Euclidean) distance. Therefore, in the graph layout, related nodes are in close proximity. Additionally, weighted adjacency matrices were calculated using edge incidence value and visualized in a weighted adjacency matrix (Figs 1b, d and 2b, d).

### Random sampling of orthogroups

To account for stochastic variation in orthologous gene clustering and to measure the efficiency of separation of genomes based on a defined set of virulence orthogroups, 312 orthogroups (i.e. the same number as the virulence orthogroups) were randomly subsampled (×100) from the complete set (*n*=9281) and a directed graph was produced for each subsampled set of orthogroups. The mean weight of each node, with 95 % confidence intervals, was measured for each subsampled orthogroup set. The resultant subsampled orthogroups were used to measure if virulence nodes were within or outside random variation.

### Annotation of plant pathogenicity genes

Virulence genes within study organisms were annotated using the VFDB as described above. Phytopathogenic virulence genes were identified from resultant VFDB functional annotations, these were: core genes for the T2, T3, T4, T6 secretion systems, flagella and pili, and T3SS effectors. The Hop and Avr nomenclature scheme was preferred; where effectors were named under an alternative schema the Hop and Avr name was adopted. The CAZy [48] and KEGG databases were used to annotate PCWDEs. A presence/absence chart was created using ggplot2 v3 and includes partial encoding of secretion systems and flagella (partial encoding means that there will be some genes present for a secretion system or flagellum but not the complete set, e.g. nine genes are required for a complete T3SS). The resultant data were compared by a chi-squared test to find similarities/differences between bacteria using counts of virulence gene categories, i.e. PCWDEs, harpins, effectors, T2SS, T3SS, T4SS, T6SS, flagella and pili. Partial encoding was not included in the chi-squared test and was changed to absent.

## Results and discussion

Genomes of *G. quercinecans* strains FRB124 and N78, and *B. goodwinii* FRB171 were generated from the 2nd generation Illumina MiSeq sequencing platform and assembled into 90, 128 and 129 contigs, respectively (Table 1). The 3rd generation Pacific Biosciences RSII platform generated data for *L. britannica* and *R. variigena*, which were assembled into one and two contigs, respectively. Furthermore, previously published genomes of *G. quercinecans*, *B. goodwinii* and *R. victoriana* were included in the analysis (Tables 1 and 2).

The taxonomy of the order *Enterobacteriales* has received significant attention and includes plant pathogens, commensals and mutualists [49]. Many consistently isolated bacterial species from the AOD pathobiome belong to the order *Enterobacteriales*, and these bacteria also dominate the lesion microbiome [3]. Therefore, the object of this study was to identify the pathogenic potential and function of bacterial members of the AOD pathobiome that belong to the order *Enterobacteriales*. This was achieved by first analysing all orthogroups from a select group of bacterial genomes (containing AOD pathobiome bacteria, plant pathogens, commensals and mutualists), secondly identifying virulence orthogroups from the complete set of orthogroups and finally annotating the phytopathogenic virulence gene homologues within virulence orthogroups. Thus, here, pathogenic potential is based on the genetic ability of a bacterium to cause disease derived on the above model [30, 50]. Orthogroup identification and subsequent separation based on shared orthology used in the first and second analyses provide an unbiased quantitative model for disentangling bacterial phytopathogenic potential. The third analysis method uses a defined set of known bacterial phytopathogen genes to characterize ecofunctional groups of bacteria. Using a combination of these methods is important, as orthology analysis provides a broad overview of pathogenic potential but does not identify idiosyncratic phytopathogenic gene homologues.

For orthology analysis, bacterial genes from all study organisms were clustered into groups sharing a common ancestor; these shared genes are orthologues and the clusters are orthologous groups (orthogroups) and totalled *n*=9281 from the study organisms (*n*=29). For example, orthogroup 1, a chemotaxis-related group, contains a total of 612 orthologues which all descend from a common ancestral gene. Orthogroup 1 is represented in 26 of the 29 study organisms. Within orthogroup 1 *B. alni* has 19 genes whereas *G. quercinecans* FRB97 has no genes. The orthogroup set (*n*=9281) was used throughout this study to identify the pathogenic potential of organisms isolated from the AOD pathobiome.

### Orthologous separation of bacterial genomes

Fig. 1(a) shows all orthogroups, in a disconnected graph, where the major graph component comprises 27 nodes, with two isolated edgeless nodes, *Bacillus licheniformis* and *Xylella fastidiosa*. Fig. 1(b) shows the relationships between orthogroups in all study organisms and is visualized through a weighted adjacency matrix, where genomes or nodes that share orthogroups have a higher edge incidence reflected through increased shading. For example, the three strains of *B. goodwinii* are shaded black indicating that they share many of the same orthogroups (e.g. *B. goodwinii* FRB141 – *B. goodwinii* FRB171 have a weighted edge of 3180), whereas *X. fastidiosa* is lightly shaded throughout the matrix sharing a low edge incidence with all other bacteria, with only marginally heavier weighting towards the genus *Xanthomonas* (e.g. *X. fastidiosa – G. quercinecans* FRB97 has a weighted edge of 1111, whereas *X. fastidiosa – Xanthomonas campestris* has a weighted edge of 1430). Graphs were drawn using a force directed algorithm, where isolated nodes such as *Xylella fastidiosa* are repulsed and connected nodes are attracted; the theoretical distance (which is measured using shared orthogroups among study organisms) is related to geometric distance in the drawing [51].

### Orthologous separation of virulence genes amongst bacterial genomes

Fig. 1(c, d) shows virulence orthogroups shared amongst all study organisms. Virulence orthogroups (*n*=312) were extracted from the identified whole genome orthogroups by firstly aligning to the VFDB and secondly through orthologous inference of the aligned virulence genes. Fig. 1(c) shows an undirected, disconnected graph (network) with a major graph containing 26 connected nodes, and three isolated nodes. The major graph consists of necrotrophs (soft-rot *Pectobacteriacae*, SRP), hemibiotrophs (*Pseudomonas syringae* pv. *syringae*), a biotrophic pathogen (*Agrobacterium tumefaciens*), a saprophyte (*Erwinia bilingiae*) and plant growth promoting rhizobacteria such as *Rhizobium leguminosarum*, *Herbaspirillum seropedicae* and *Gluconacetobacter diazotrophicus* (Fig. 1c). The major graph is similar to Fig. 1(a) with the notable exceptions of *G. quercinecans* N78 which is connected with comparatively low edge incidence (degree=2) to the major graph in Fig. 1(a) and forms an isolated node in Fig. 1(c) (degree=0). *G. quercinecans* N78 was isolated from Spain and is a species which has high genetic diversity, explaining why it lacks the orthologous relationship of the strains isolated in Britain (*G. quercinecans* FRB97 and FRB124) [52]. Furthermore, the relationships of bacteria isolated from AOD lesions is shown in Fig. 2(a, b). *G. quercinecans* was recently described largely based on isolates found in Britain, and it is possible that *G. quercinecans* N78 represents a different species, as the 16S phylogeny separated the Spanish strains from the British strains [5]. Further isolated nodes in Fig. 1(c) include *Methylobacterium mesophilicum* (degree=0), but which is connected to the major graph of Fig. 1(a) (degree=7), which would be expected as the bacterium is a mutualist. Similarly, *Rhizobium leguminosarum* bv. *trifoli* and *viciae* have a degree of 7 and 11 in the major graph of Fig. 1(a) but are mutualists which lack both T2 and T3 secretion systems and therefore both have a reduced degree of 2 in Fig. 1(c), with only shared edges to each other and *Agrobacterium tumefaciens,* which brings them into the major graph. *Ralstonia solanacearum* is connected to the major graph with a degree of 1, inferring divergence from other plant pathogens in the study, probably due to an idiosyncratic biotrophic pathogenicity mechanism, characterized by a non-necrotizing mode of action [53]. By injecting T3 effectors into host cells *R. solanacearum* halts expression of salicylic acid mediated defences and multiplies to high cell densities causing occlusion of the xylem vessels, leading to non-necrotic bacterial wilt on over 200 phylotype specific hosts [54–56]. This non-necrotizing mechanism is an example of plant–pathogen co-evolution, enabling *R. solanacearum* to avoid immune detection, but differentiating this pathogen from those using a necrotrophic mode of action.

**Fig. 3.**
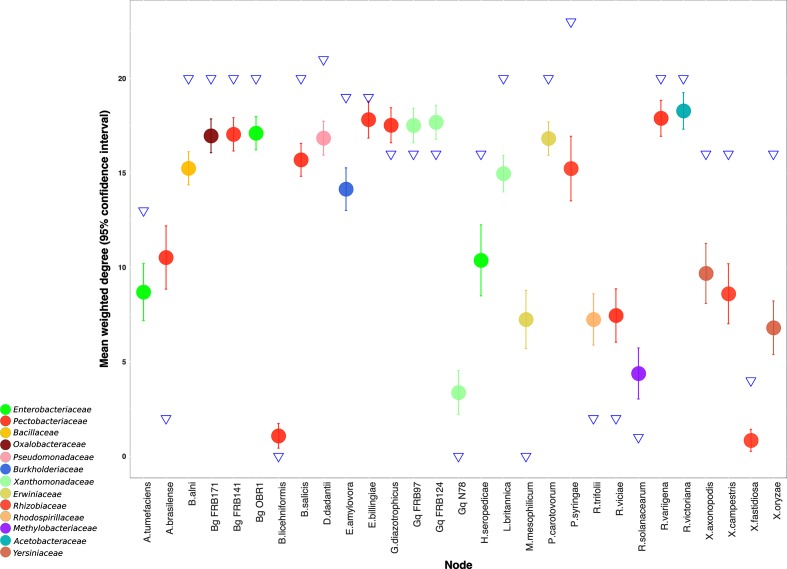
Mean weighted degree of all orthogroups from random subsampling of study organisms. The mean weighted degree of each bacterium is represented by a circle with 95 % confidence intervals measured using random subsampling. The degree of virulence orthology inference for each bacterium is represented by a blue triangle. Nodes are coloured by bacterial family. Gq=*Gibbsiella* *quercinecans*, Bg=*Brenneria* *goodwinii*.

**Fig. 4.**
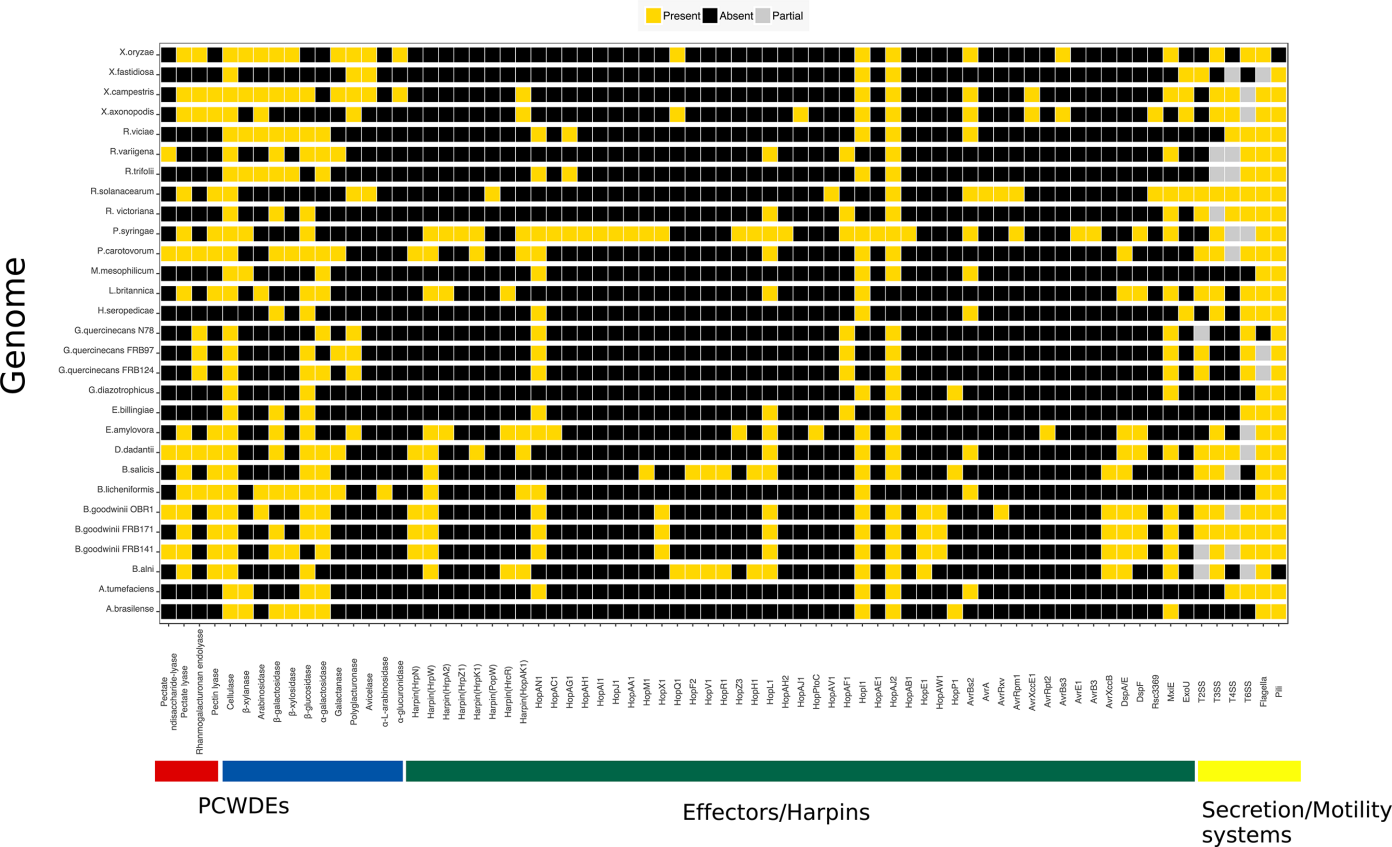
Presence/absence chart of genome encoded phytopathogenic virulence factors within all study organisms. Gene/gene systems present=gold, absent=black, partial=grey: partial only applies to secretion/motility systems. Genes are separated into three categories (*x*-axis): PCWDEs (plant cell wall degrading enzymes)=red/blue, PCWDEs are subdivided into pectin lyases (red) and glycoside hydrolases (blue); effectors/harpins=green; secretion/motility systems=yellow. The colour of bacterial names is separated by family.

There is a broad pattern in the orthogroup inference classification as pathogens have an increased or equal degree among virulence orthogroups whereas non-pathogens have a reduced degree in the virulence orthogroups (Fig. 3, Tables 1 and 2). This shows that pathogens have increased connectivity to the major virulence graph (e.g. *Dickeya dadantii* has a degree of 19 in Fig. 1a, which is increased to 21 in Fig. 1c) whereas non-pathogens have decreased connectivity (e.g. *Azospirillum brasilense* has a degree of 20 in Fig. 1a, but this is reduced to 2 in Fig. 1c). *B. goodwinii* and *L. britannica* follow the trend of phytopathogens in having an increased degree of orthologous genes in the virulence orthogroups (Fig. 3, Tables 1 and 2), probably due to the presence of a T3SS, harpins, effectors and pectin lyases. This is also true for *X. fastidiosa* which forms an isolated node in Fig. 1(a) but connects to the three *Xanthamonas* species in the major virulence graph and has a degree of 4, inferring that *X. fastidiosa* has a stronger relationship with virulence gene vs. genome-wide orthology within the study bacteria. The only outlier within Fig. 3 is *P. syringae* pv. *syringae*, which has the highest degree in both graphs, but which decreases from 25 to 23 degrees in the virulence graph (Fig. 1c). This may be due to the phylogenetic position of *P. syringae* pv. *syringae* within the order *Gammaproteobacteria*, where it shares close relationships with many of the study organisms giving a high representation of *P. syringae* pv. *syringae* genes among orthogroups [57]. A decreased degree among virulence orthologues of *P. syringae* pv. *syringae* may reflect the higher selective pressure on virulence genes, which means that genes which were orthologous to those in other species no longer have a common function and are no longer orthologues [58]. Some of the study organisms such as *Erwinia bilingiae* and *E. amylovora* demonstrated little change between graphs (*E. billingiae* had a reduced degree in the major virulence graph from 22 to 19, whereas *E. amylovora* increased in the major virulence graph from 15 to 19). *E. billingiae* occupies a genus that contains plant pathogens, including *E. amylovora*. A clear distinction between *E. billingiae* and *E. amylovora* is demonstrated in the genome-encoded direct virulence factors: the T3SS and multiple associated effectors, which are present in the pathogen *E. amylovora*, but absent from the saprophyte *E. billingiae* (Fig. 4).

### Description of *B. goodwinii*, *G. quercinecans*, *Rahnella* species and *L. britannica* encoded virulence factors

Annotation of phytopathogenic virulence gene homologues within all study organisms revealed a high level of variation using a chi-squared test of association (*P*=8.47×10^−11^). All known *Brenneria* species are tree pathogens and the evidence presented here adds *B. goodwinii* to the cohort of phytopathogenic bacteria within the genus *Brenneria* [49]. Phytopathogenic virulence homologues encoded within *B. goodwinii* were compared with other study organisms to determine significant associations (Fig. 4), revealing that *B. goodwinii* is more closely associated with necrotrophs than hemibiotrophs. *B. goodwinii* has a closer association to the necrotrophic SRP (*P*=0.83 association with *P. carotovorum* and *P*=1 association with *D. dadantii*) than the hemibiotroph *P. syringae* pv. *syringae* (*P*=0.01). This method of virulence gene annotation and testing for significant differences is an *in silico* replacement for the pathogenic potential which is measured using model organisms [30]. However, an idiosyncratic pathogenicity mechanism which is similar to both the SRP and *P. syringae* pathovars is possible, due to the encoded T3SS, harpins and effectors – typical of hemibiotrophs – and PCWDEs – typical of necrotrophs.

The T3SS is critical to the virulence of numerous animal and plant pathogens [15]. For example, *﻿Salmonella enterica subsp*. enterica** serovar Typhimurium and *P. syringae* pathovars use the T3SS to manipulate host defences and metabolism, allowing bacteria to colonize and maintain a supply of nutrients [59, 60]. *B. goodwinii* and *L. britannica* have a similar *in silico* pathogenic potential (*P*=0.19); both species have a complete T3SS (i.e. nine core genes which comprise the T3 nanomachine), as do the non-AOD-related phytopathogens *B. alni* and *B. salicis*. Genomic annotations in this study identified numerous homologues of effectors within three *B. goodwinii* strains: HopAN1, HopX1, HopL1, HopI1, HopAJ2, HopA1, HopAW1, AvrXccB, DspA/E, DspF, MxiE and AvrAxv (the last encoded within *B. goodwinii* ORB1 only). The *hrpN* harpin gene is key to the virulence of *E. amylovora* and secretes the DspA/E effector from the intercellular apoplastic space into intracellular space of a host cell; both *hrpN* and *dspA/E* are encoded within *Brenneria* species and *L. britannica* [61–63]. Crucially, *hrpN*, *dspA*/E, *hopX1* and *hopE1*, originating from *B. goodwinii*, were significantly differentially upregulated in a metatranscriptomic analysis of AOD lesions, revealing the prevalence of *B. goodwinii* within the lesion environment and the importance of T3SS and effectors to AOD tissue necrosis [7]. Within the *B. goodwinii* effector repertoire are virulence factors, homologues of which are key to disease causation in bacterial phytopathogens; furthermore those upregulated in necrotic oak tissue and originating from *B. goodwinii* (*dspA*/E, *hopX1*, *hopE1*, *hopAW1* and *avrXccB*) are key targets for future knock out and expression studies.

Orthologous virulence gene clustering positioned *G. quercinecans* most closely with saprophytes and plant pathogens (Fig. 1d). This is consistent with previous findings where *G. quercinecans* inoculated onto oak logs (analogous to an immunocompromised host) resulted in lesion formation [3]. A previous study concluded that *G. quercinecans* is a saprophyte as it was isolated from decaying wood, and has high genetic diversity, although the authors acknowledge that the definitions of saprophytes compared to pathogens are opaque [64]. This is especially true within an immunocompromised host such as predisposed oak trees, where the host has an altered microbiome and defence responses [3]. *Rahnella* species were included in the major graph in Fig. 1(a, c) but lack a T3SS or major PCWDEs and have a closely associated *in silico* pathogenic potential to *G. quercinecans* (*P*=0.68). Similarity of virulence orthogroups between *G. quercinecans*, *Rahnella* species and other bacteria isolated from AOD lesions shows that *G. quercinecans* and *Rahnella* species have separate repertoires of virulence gene orthologues (Fig. 2c, d). Unlike *G. quercinecans* (rarely found outside of the AOD lesion pathobiome), *R. victoriana* was found consistently in AOD symptomatic, and non-symptomatic trees, and therefore it may be inferred that *R. victoriana* and *R. variigena* are saprophytes [3]. However, as has been previously discussed, saprophytes can cause disease under requisite circumstances. Due to the lack of a T3SS and nature of AOD tissue necrosis, the most probable virulence mechanisms of *G. quercinecans* and *Rahnella* species would be through the release of PCWDEs and persistence factors. However, they are not significantly associated with the SRP (both *Rahnella* species and *G. quercinecans* have *P≤*0.05, with the SRP; Fig. 4) due to the high number of PCWDEs and specifically pectin lyase genes typically found in necrotrophs such as the SRP, which makes them such devastating pathogens [65]. However, *G. quercinecans* and *Rahnella* species may not have primary pathogen functionality but instead fulfil an analogous role to that of *Pantoea agglomerans*, *E. toletana* and *E. oleae* in olive knot disease [66]. These non-pathogenic bacteria co-operate with *Pseudomonas savastanoi* pv. *savastanoi* to modulate disease severity [67]. This theory is supported by the non-fastidious nature of *G. quercinecans* and *Rahnella* species, which are more robust and cultivatable than the more labile *B. goodwinii* [3]; one possibility is that *G. quercinecans* and *Rahnella* colonize a declining oak tree prior to *B. goodwinii*, thereby creating an environmental niche for *B. goodwinii* to colonize and express virulence factors.

Classification of pathogenic potential *in silico* presents challenges as evidence of host damage is not available. Furthermore, the prevalence of virulence genes within symbionts hinders automated classification [68]. Encoded virulence factors within symbionts are explicable as all symbionts have to colonize, persist and reproduce – necessitating virulence-like genes and systems [69]. For example, the T3SS is not confined to pathogens, and also functions as a host interaction component, albeit rarely [70]. The T3SS has a dual role within the genus *Herbaspirillum* which includes T3SS encoding symbiotic and pathogenic species. *Herbaspirillum seropedicae* is a diazotrophic, mutualistic species with a functional T3SS and the requisite nine core genes encompassing the Hrp conserved operon (hrcCNQJSTRUV) (Fig. 4). Notably, *H. seropedicae* has comparatively few effectors, and those present, i.e. HopJ2, HopI and HopAN1, are also found in bacteria lacking a functional T3SS (Fig. 4). The T3SS operon within *H. seropedicae* is organized identically to the pathogenic species *H. rubrisubalbicans* but has only one shared effector gene – *hopAN1* [71]. The *hopAN1* effector gene is present in plant pathogen strains such as *P. syringae* pv. *syringae* B728a and the non-T3SS encoding symbionts *Methylobacterium mesophilicum* SR1, *E. billingiae* Eb661, and three strains of *G. quercinecans:* N78, FRB97 and FRB124 (Fig. 4). This is anomalous as the prevalence of these effectors within a wide range of non-T3SS encoding symbiotic bacteria implies that they are more than evolutionary remnants which are yet to be purged as energetically expensive unnecessary genes [23], but indicates a broader role, where their functionality is not related to T3 secretion. This creates ambiguity for T3SS delineation upon annotation, a difficultly which is also true for other virulence homologues. This has led to calls for more rigorous appraisal of virulence gene homologues involved in host interaction as opposed to their automated listing within the virulence arsenal [72].

### Investigating the AOD pathobiome

Distinguishing pathogens *in planta* can be confirmed by damage to host tissue [26]. Confirmatory tests of disease aetiology are typically based on fulfilment of Koch’s postulates, which necessitates that a single pathogen causes disease, on the same host or using an appropriate model organism [31]. Pathobiome research often cannot fulfil these tests, as there is often no primary pathogen, or the primary pathogen is attenuated without requisite pathobiome consortia [73, 74]. AOD research is hampered by difficulties in obtaining a suitable experimental host, as using oak trees or oak logs is limited by availability, and ethical considerations of using numerous long-lived organisms. Previously, oak logs were used to mimic a predisposed oak host and suitable pathobiome consortia were inoculated into the phloem and sapwood tissues, but a considerable time period must elapse before the outer bark can be removed to visualize vascular tissue damage [3]. Without considerable resources and time, the replicate scale of these experiments is low, which makes standard pathology methods such as gene knockout experiments to validate bacterially mediated necrosis outside of typical research cycles.

## Conclusions

WGS data provides key information that can infer pathogenicity. Data presented here compares orthologous genes between canonical phytopathogens, non-pathogenic symbionts and AOD lesion microbiota. The aim of this study was to investigate the pathogenic potential and functional capabilities of AOD lesion microbiota. Results reveal that *B. goodwinii*, *G. quercinecans, R. victoriana*, *R. variigena* and *L. britannica* have the genome encoded potential to cause disease, but even with a requisite gene set the outcome of host–microbe interactions is inherently unpredictable due to the number of variables involved. However, within an immunocompromised host, interactions with bacteria containing a substantial pathogenic potential or infection with a high bacterial load may have deleterious outcomes for the host, which in a healthy host or with a low bacterial inoculum may have had a benign outcome. The terms pathogen, saprophyte and commensal are not useful in this scenario. A more accurate summation is that bacteria have a pathogenic potential and the outcome of host–bacteria interactions is dependent on multiple factors.

Notably, *B. goodwinii* and *L. britannica* have a T3SS and associated harpins and effectors, giving these organisms the genomic potential to manipulate the plant host and cause tissue necrosis. However, empirical data reveal that *B. goodwinii* was consistently isolated from the pathobiome, whereas *L. britannica* was rarely isolated [3]. *G. quercinecans*, *R. variigena* and *R. victoriana* encode pathogenicity genes, but have a lower genome encoded pathogenic potential than *B. goodwinii* and *L. britannica*. However, they may be able to cause pathogenicity in given scenarios as has been proven for *G. quercinecans* using oak log infection assays [3]. The role of *G. quercinecans*, *R. variigena* and *R. victoriana* is perhaps analogous to that of *Erwinia* species and *Pantoea agglomerans*, which act as pathobionts within the olive knot pathobiome, enhancing disease caused by ﻿*Pseudomonas savastanoi* pv. *savastanoi* [67]. Empirical ecological evidence combined with the present study shows *B. goodwinii* as a key causal agent of AOD and *L. britannica* as an infrequent component of the pathobiome which is capable of necrosis.

The polymicrobial nature of AOD challenges traditional orthodoxies reliant on Koch’s postulates and characterization of single primary pathogens as a diagnostic measure of disease. Within AOD, multiple species interact to cause disease as pathobiome constituents without an apparent primary pathogen. Urgent research and control measures are required as AOD is a growing threat to oak in the UK, the European mainland and further afield. Furthermore, a new paradigm is required as an addendum to Koch’s postulates, specifying the requisite steps for proving pathobiome-mediated disease. Overall, this study provides computational analysis of AOD pathobiome consortia with resultant data adding to empirical ecological evidence implicating primarily *B. goodwinii* as an essential virulence component within the AOD lesion pathobiome.

## Data bibliography

1. The complete and annotated genome of the following bacteria can be found in GenBank under the listed accession numbers: *Agrobacterium tumefaciens* Ach5 (CP011246), *Azospirillum brasilense* Sp7 (CP012914), *Bacillus licheniformis* ATCC 14580 (NC_006270), *Brenneria goodwinii* OBR1 (CGIG00000000), *Dickeya dadantii* 3937 (NC_014500), *Erwinia amylovora* CFBP1430 (NC_013961), *Erwinia billingiae* Eb661 (NC_014306), *Gluconacetobacter diazotrophicus* PA1 5 (NC_011365), *Herbaspirillum seropedicae* Z67 (CP011930), *Methylobacterium mesophilicum* SR1.6/6 (ANPA01000003), *Pectobacterium carotovorum* subsp. *carotovorum* PC1 (NC_012917), *Pseudomonas syringae* pv. *syringae* B728a (NC_007005), *Ralstonia solanacearum* GM1000 (NC_003295), *Rhizobium leguminosarum* bv. *trifolii* WSM1689 (CP007045), *Rhizobium leguminosarum* bv. *viciae* 3841 (NC_008380), *Xanthomonas axonopodis* Xac29-1 (NC_020800), *Xanthomonas campestris* ICMP 21080 (CP012145), *Xanthomonas oryzae* pv. *oryzae* MAFF 311018 (NC_007705), *Xylella fastidiosa* Hib4 (NZ_CP009885).
